# Periprocedural anticoagulation in atrial fibrillation: Update on electrical cardioversion and ablation

**DOI:** 10.1007/s12471-018-1119-z

**Published:** 2018-05-09

**Authors:** S. P. G. van Vugt, M. A. Brouwer

**Affiliations:** Department of Cardiology, Radboud University Medical Center, Nijmegen, The Netherlands

**Keywords:** Atrial fibrillation, Electrical cardioversion, Catheter ablation, Vitamin K antagonist, Non-vitamin K oral anticoagulant

## Abstract

In this manuscript, we discuss the most important changes in the field of anticoagulant treatment in patients with atrial fibrillation in the setting of electrical cardioversion or catheter ablation. Moreover, we provide practical guidance as well as information on daily practice.

## Electrical cardioversion for atrial fibrillation

For successful restoration of sinus rhythm in patients with atrial fibrillation (AF), electrical cardioversion (ECV) is a quicker and more effective strategy than pharmacological cardioversion, with the highest success rates in case of pretreatment with antiarrhythmic drugs [1–5]. However, the success rate should be weighed against the involved disadvantage of sedation, the need of fasting, and the procedure-related increase in stroke risk [6].

After the landmark AF trials with the non-vitamin K oral anticoagulants (NOACs), several randomised trials on ECV have been published [7–9]. The periprocedural use of the new agents is now addressed in more detail in the 2016 European AF guidelines [3]. For patients in whom cardioversion is performed within 48 hours of AF onset, the recommendations with regard to periprocedural anticoagulation have become less liberal over the years [3, 10]. Notably, preprocedural anticoagulation is now recommended for all patients, irrespective of risk factors for stroke. The same holds true for the 4‑week postprocedural anticoagulation regimen. These recommendations pertain to both chemical and electrical cardioversion [3].

In addition to a short update of the most important changes, and a review of the NOAC-related regimens, we provide some practical guidance and information on daily practice of anticoagulation in the setting of cardioversion in Europe.

**Figure:**
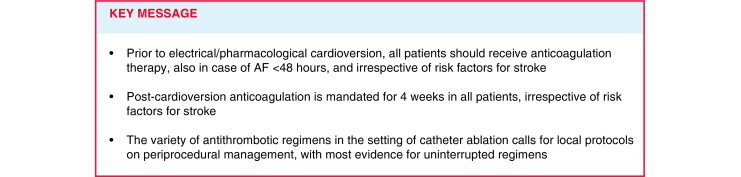

### The evidence

#### Periprocedural anticoagulation

**Fig. 1:**
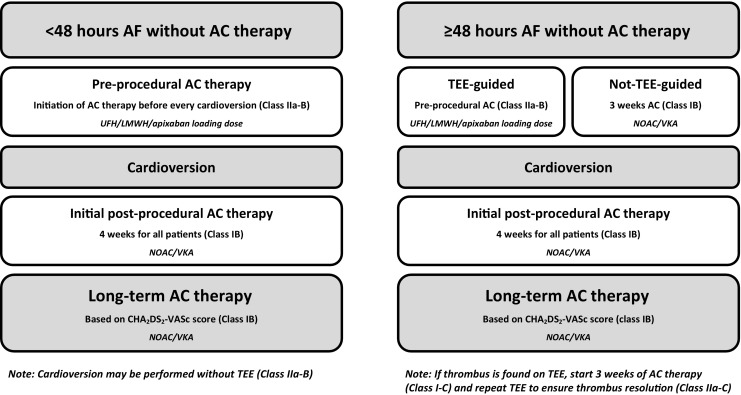
Pericardioversion anticoagulation: key recommendations in the ESC 2016 AF guidelines (*ESC* European Society of Cardiology, *AF* atrial fibrillation, *AC* anticoagulation, *UFH* unfractionated heparin, *LMWH* low-molecular-weight heparin, *NOAC* non-vitamin K oral anticoagulant, *VKA* vitamin K antagonist, *CHA*_*2*_*DS*_*2*_*-VASc score* Congestive heart failure, Hypertension, Age ≥ 75 [doubled], Diabetes, prior Stroke [doubled]—Vascular disease, Age 65–74, Sex category, *TEE* transoesophageal echocardiography)

Whereas for AF of more than 48 hours of duration no important changes have been reported, a stricter regimen has been introduced for anticoagulation therapy prior to ECV in the setting of short duration AF (Fig. 1; [3]). Previously, patients with AF < 48 hours were triaged for preprocedural heparin based on their stroke risk profile, but the large FinCV registry on 5116 cardioversions reported a clear increase in the risk of stroke when AF duration exceeded 12 hours. This also applied to patients with CHA_2_DS_2_-VASc [Congestive heart failure, Hypertension, Age ≥ 75 [doubled], Diabetes, prior Stroke [doubled]—Vascular disease, Age 65–74, Sex category] scores of 0–1 (Table 1; [11]). In the subset of 2,481 patients with neither preprocedural heparin nor subsequent anticoagulation therapy, the risk of thrombo-embolic events for CHA_2_DS_2_-VASc 0–1 was at least as high as for patients with CHA_2_DS_2_-VASc 2 [12]. Stroke rates were markedly lower when on antithrombotic therapy, with no events in CHA_2_DS_2_-VASc 0–1 in those on treatment versus 0.4% to those without [13]. Whereas in the 2010 European AF guideline preprocedural heparin was recommended based upon the estimated risk profile for stroke (Class I-B), the latest guidelines advocate preprocedural anticoagulation for all patients with AF < 48 hours, regardless of stroke risk factors (Class IIa-B) [3, 10]. In the context of stroke rates of 0.5–1% in the month after cardioversion, a properly powered randomised controlled trial is not realistic. This is why registry data currently represent the best available evidence on the benefit of standard anticoagulation prior to the ECV [13, 14]. Similar restrictions apply to the evidence used to change recommendations on the post-cardioversion antithrombotic regimen.

**Table 1:** Cardioversions and thrombotic complications in patients with acute atrial fibrillation in the FinCV Study

	Duration of atrial fibrillation					
Patients without anticoagulation therapy	<12 hours (*n* = 2440)		12–24 hours (*n* = 1840)		24–48 hours (*n* = 836)	
	*N* (%)	95% CI	*N* (%)	95% CI	*N* (%)	95% CI
Stroke/systemic embolism	8 (0.3)	0.1–0.6%	21 (1.1)	0.7–1.6%	9 (1.1)	0.4–1.8%

*CI* confidence interval

#### Postprocedural anticoagulation

Another large registry, reporting outcome in 16,274 patients from Denmark, demonstrated that the risk of no anticoagulation therapy was similar for patients with a CHA_2_DS_2_-VASc score of 0–1 (odds ratio [OR] 2.21; 95% confidence interval [CI] 0.79–6.77) and those with a CHA_2_DS_2_-VASc score of ≥ 2 (OR 2.41; 95% CI 1.46–3.95) [14]. Whereas previously the recommendation of a 4-week anticoagulation strategy after ECV was restricted to patients with risk factors for stroke, these and other observations support the current advice to prescribe oral anticoagulation therapy after ECV in all patients [3, 10, 13–16]. First, the risk of stroke increases after cardioversion, not only for patients with a high, but also for patients with a very low CHA_2_DS_2_-VASc score. For patients with a CHA_2_DS_2_-VASc score of 0–1, with a yearly stroke risk of up to 1.3%, the observed 30-day stroke rate post-cardioversion is about 0.5%. In the context of the estimated 1‑year stroke risk, this represents a marked increase [6, 10]. Even in patients with AF lasting less than 48 hours, the 30-day stroke risk has been reported to be considerably higher than the estimated 1‑month risk as would be expected based upon the annual stroke risk [3, 13]. In short, when a patient is referred for ECV, we predispose the patient to a higher risk of stroke, even when using the recommended postprocedural anticoagulation [6, 13]. About half of these strokes occur within 2–5 days [12, 17, 18]. Importantly, the pathophysiological mechanism of the thrombo-embolic events is multifactorial, and even more complex than during the natural course of AF [15]. Preprocedural duration of AF, CHA_2_DS_2_-VASc score and parameters that indicate atrial function recovery have all been associated with the risk of thrombotic complications [11, 13, 15, 19]. Importantly, the effect on electrical atrial remodelling not only applies to electrical, but also to pharmacological cardioversion [19]. These findings support the revisions in the present guideline, that endorse post-cardioversion anticoagulation in all patients with AF < 48 hours, irrespective of risk factors (Class I-B). As for anticoagulation beyond the 4‑week interval, no changes have been reported and the indication should be made based upon the CHA_2_DS_2_-VASc score (Class I-B).

#### Unfractionated heparin (UFH), low-molecular-weight heparin (LMWH)

Despite the focus on a stricter anticoagulation regimen, also in AF < 48 hours, little information is provided on the recommended dose of preprocedural heparin. In summary, most studies report for UFH a dose of 70–80 IU/kg, with a total bolus of about 5000 IU [20, 21]. The effect of UFH sets in quickly (within half an hour) and lasts for about 1–2 hours. As for enoxaparin, a subcutaneous dose of 1 mg/kg twice daily is most commonly reported; it has a slower onset of action (3–5 hours after subcutaneous injection) [20, 22, 23]. Please note that a vitamin K antagonist (VKA) prescribed as a post-cardioversion anticoagulation strategy in anticoagulation-naive patients requires bridging with enoxaparin or UFH until the international normalised ratio (INR) has reached the target range. As stated before, ECV by itself creates a pro-thrombotic state with events often seen within the first 2–5 days and thus early, optimal anticoagulation is a prerequisite [12, 17, 18].

#### NOACs versus VKA

Appreciating the low event rates in the setting of ECV, properly powered trials are a logistical challenge. Dabigatran was the first NOAC mentioned in the guidelines for the indication of ECV, based on a subgroup analysis from the RE-LY trial [24, 25]. Currently, subgroup analyses from all four large AF trials are available and several observational studies have been reported (Table 2; [24, 26–33]). In addition, randomised trials for rivaroxaban, edoxaban and apixaban have been completed [7–9]. Given their direct onset of the anticoagulant effect, NOACs are also promising drugs to substitute preprocedural heparin/LMWH. The only randomised trial that provides data on patients with AF < 48 hours is the EMANATE study, in which a subset of 342 patients received a loading dose of apixaban, administered at least two hours before cardioversion, the majority under guidance of transoesophageal echocardiography (TEE) [9]. Thus, most evidence of NOACs in the setting of cardioversion pertains to AF with a duration of > 48 hours (Table 3). To expedite early cardioversion, there was a minimal pretreatment duration with rivaroxaban of 1-5 days, and about 3 days with edoxaban (Table 4; [7, 8]). For apixaban, a 10 mg loading dose with subsequent cardioversion was possible (2 hours after intake), in other cases at least five regular doses of apixaban were required before cardioversion [9]. A meta-analysis on randomised data and information obtained from subgroup analyses demonstrates similar efficacy (0.4%) and safety (0.6%) between NOACs and VKA and thereby support the use of NOACs in the setting of ECV (Tables 5 and 6; [34]).

**Table 2:** Post hoc analyses on NOAC and cardioversion

Trial	RE-LY	ROCKET-AF	ARISTOTLE	ENGAGE AF-TIMI 48
NOAC	Dabigatran	Rivaroxaban	Apixaban	Edoxaban
Patients	*n* = 1270	*n* = 285	*n* = 540	*n* = 365*
Number of CVs	1983	375	743	632
On randomised treatment	80%	87%	84%	100%
TEE-guided CVs	21%	n/a	27%	n/a
Follow-up duration	30 days	30 days	30 days	30 days

*AF* atrial fibrillation, *TIMI* Thrombolysis in Myocardial Infarction, * NOAC* non-vitamin K oral anticoagulant, *CVs* cardioversions, *TEE* transoesophageal echocardiography, *n/a* not available

*including 111 patients on low-dose edoxaban (30 mg/15 mg)

**Table 3:** Randomised trials on NOAC and cardioversion

Trial	X-VeRT	EMANATE	ENSURE-AF
Comparison	Rivaroxaban vs. VKA	Apixaban vs. heparin/warfarin	Edoxaban vs. enoxaparin/warfarin
Patients	*n* = 1504	*n* = 1500	*n* = 2199
AF duration	≥48 h	<48 hours and ≥48 hours^a^	≥48 h
Treatment strata	Early or delayed	Imaging or no imaging	TEE or non-TEE
Stratum 1	Early (58%)	Imaging (57%)^b^	TEE (54%)
≥3 weeks OAC	47%	n/a	n/a
TEE-guided CV	Rivaroxaban 67%, VKA 65%	100%^c^	100%
Stratum 2	Delayed (42%)	No imaging (43%)^b^	Non-TEE (46%)
≥3 weeks OAC	100%	n/a	100%
TEE-guided CV	Rivaroxaban 8%, VKA 14%	0%	0%

*AF* atrial fibrillation, *CV* cardioversion, *NOAC* non-vitamin K oral anticoagulation, *OAC* oral anticoagulation,* TEE* transoesophageal echocardiography,* VKA* vitamin K antagonist

^a^ 2/3 of patients had new onset AF

^b^optional loading dose of 10 mg apixaban ≥ 2 h before cardioversion

^c^TEE or computed tomography

**Table 4:** Anticoagulation strategies in randomised trials on NOAC and cardioversion

Trial	X-VeRT		EMANATE		ENSURE-AF	
Comparison	Rivaroxaban vs. VKA		Apixaban vs. heparin/warfarin		Edoxaban vs. enoxaparin/warfarin	
Treatment strategy	TEE	No TEE	Imaging	No imaging	TEE	No TEE
Pre-procedural anticoagulation	1–5 days	3–8 weeks	~60% 3-4 days^a^; ~40% 2-3 weeks	~25% 3-4 days^a^; ~75% 4 weeks	≤3 days	≥3 weeks
Post-procedural anticoagulation	42 days		30 days		28 days	

*AF* atrial fibrillation, *VKA* vitamin K antagonist, * TEE* transoesophageal echocardiography, *NOAC* non-vitamin-K anticoagulant

^a^loading dose of apixaban

**Table 5:** Efficacy and safety outcomes in post hoc analyses on NOAC and cardioversion

Trial	RE-LY	ROCKET-AF	ARISTOTLE	ENGAGE AF-TIMI 48
Comparison	Dabigatran vs. warfarin	Rivaroxaban vs. warfarin	Apixaban vs. warfarin	Edoxaban vs. warfarin
SSE^a^	11 (0.6%)	2 (0.7%)	0 (0%)	0 (0%)
NOAC vs. VKA	7 (0.5%) vs. 4 (0.6%)	n/a	–	–
Major bleeding^a^	19 (1.0%)	n/a	2 (0.2%)	0 (0%)
NOAC vs. VKA	15 (1.1%) vs. 4 (0.6%)	n/a	1 (0.3%) vs. 1 (0.2%)	–

*AF* atrial fibrillation, *SSE* stroke or systemic embolism*, n/a* not available,* NOAC* non-vitamin-K oral anticoagulant, *TIMI* Thrombolysis in Myocardial Infarction, *VKA* vitamin K antagonist

^a^After 30 days

**Table 6:** Efficacy and safety outcomes in randomised trials on NOAC and cardioversion

Trial	X-VeRT	EMANATE	ENSURE-AF
Comparison	Rivaroxaban vs. VKA	Apixaban vs. heparin/warfarin	Edoxaban vs. enoxaparin/warfarin
Primary efficacy endpoint^a^	10 (0.7%)	6 (0.4%)	16 (0.7%)
NOAC vs. VKA	5 (0.5%) vs. 5 (1.0%)	0 (0%) vs. 6 (0.8%)	5 (0.5%) vs. 11 (1.0%)
Primary safety endpoint^b^	10 (0.7%)	33 (2.2%)	27 (1.2%)
NOAC vs. VKA	6 (0.6%) vs. 4 (0.8%)	14 (1.9%) vs. 19 (2.5%)	16 (1.5%) vs. 11 (1.0%)

*AF* atrial fibrillation, *NOAC* non-vitamin-K oral anticoagulant, *VKA* vitamin K antagonist

^a^X-VeRT: stroke/systemic embolism (SSE), transient ischaemic attack (TIA), myocardial infarction (MI) or cardiovascular (CV) death; EMANATE: SSE; ENSURE-AF: SSE, MI or CV death

^b^X-VeRT: International Society on Thrombosis and Haemostasis bleeding scale (ISTH) major bleeding; EMANATE and ENSURE-AF: major and clinically relevant non-major bleeding

In summary, preprocedural anticoagulation for short duration AF is currently also recommended in patients without risk factors for stroke. Theoretically, the standard preprocedural administration of anticoagulation may carry a small, though somewhat higher risk of bleeding. With regard to post-cardioversion anticoagulation in all patients, this may have consequences as well, although the safety profile of NOACs is reassuring.

Importantly, for early, immediate cardioversions (for example, in case of haemodynamic instability) in anticoagulation-naive patients UFH intravenously provides an immediate anticoagulant effect. The only NOAC tested with early cardioversions is apixaban (loading dose 10 mg), but this still requires a 2-hour interval before to proceed. The other NOACs were used for at least one day before ECV was performed. As for elective cardioversions, a 3-week pretreatment period, with at least for 4 weeks of post-treatment has been studied with all NOACs.

#### Compliance

In contrast to the situation with VKA, there is no objective tool to monitor the quality of anticoagulation of the preprocedural period with NOACs. Compliance is therefore of the utmost importance, and verification of compliance on the day of the ECV is crucial. Observations in patients treated with VKA have demonstrated that complications are much more frequent in those with a suboptimal intensity at the day of the procedure [18]. Although the randomised trials with NOACs on ECV do not provide very detailed definitions on preprocedural compliance, we advocate that none of the doses should be omitted in the three days before the procedure. The general rule that compliance is considered sufficient in case of an intake of 80% or more seems too arbitrary in the setting of cardioversion, as it does not cover the scenario that two missed doses in the three days before the procedure may still render a more than 80% intake [35].

### Daily practice

From surveys in Europe, including the RHYTHM-AF registry, we have learned that our current practice of ECV has improved over the years [1, 36, 37]. The prescription of preprocedural antiarrhythmic drugs, which has been shown to improve success rates, still deserves attention [3]. Many centres experience an increase in the number of cardioversions per year, and at least 40% of the interviewed centres started implementing periprocedural use of NOACs [37]. Overall, estimates are that at present at least 25% of all cardioversions are performed on NOACs [37]. Little information is available on approaches that will ensure and control medication adherence, but as of yet the registry data on thrombo-embolic events are reassuringly low [29–32].

As for AF > 48 hours, the majority of centres (~70%) followed the mandatory 3‑week period of oral anticoagulation prior to elective cardioversion, and in the other centres a TEE-guided approach was adopted [37]. In this survey, performed before the latest guideline update, about a third of the centres already had a protocol with 4 weeks of post-cardioversion anticoagulation for AF < 48 hours, even when the embolic risk was low [37].

These surveys provide very informative insights into current developments and the areas in which we can still improve with regard to both antiarrhythmic and antithrombotic management in the setting of ECV.

## Ablation therapy for atrial fibrillation

Over the past years, invasive treatment of atrial arrhythmias has evolved, and most of the current evidence on antithrombotic therapy has been collected in the setting of cryoballoon and radiofrequency ablation therapy [38]. Both thrombotic complications and bleeding events are events related to the specific technique of the procedure, the underlying disease and periprocedural antithrombotic management.

In addition to the induced tissue damage, temperature changes with application of cryoenergy and radiofrequency also affect the thrombotic state. Moreover, in some cases, AF is converted into sinus rhythm during the procedure, which is an additional mechanism that may contribute to a higher thrombotic risk, as described earlier for cardioversions [39].

The abovementioned conditions underscore why optimal procedural anticoagulation is a prerequisite. At present, this is recommended by use of heparin and a target activated clotting time of >300 sec, although data to confirm this target are lacking. To minimise the presence of intra-atrial thrombus prior to the procedure, a period of 4 weeks of preprocedural anticoagulation has been advocated, in line with the recommendations for oral anticoagulation for ECV [39]. Historically, acenocoumarol and phenprocoumon were the drugs of choice for preprocedural anticoagulation in the Netherlands, which were discontinued prior to the procedure. Bridging with LMWH was performed before and after the ablation, followed by resumption of VKA. In the past few years, uninterrupted anticoagulation has become the standard, and the first randomised evidence for periprocedural use of NOACs has been published [40–42].

In addition to a brief review of the evidence, we will highlight a European survey on current practice, and provide some practical guidance for optimal periprocedural anticoagulation care [43].

### Peri-ablation anticoagulation—the evidence

#### VKA

After the completion of the COMPARE trial, uninterrupted use of VKA has become the standard, and bridging is not recommended anymore [40]. In this randomised study with over 1500 patients continuous use of VKA (INR 2.0–3.0) was associated with a significantly lower rate of thrombo-embolic events, and a 40–50% reduction in major bleeding as compared with a strategy with LMWH bridging [40]. Despite this improvement, the use of VKA has been associated with a disadvantage; the intensity of anticoagulation tends to fluctuate, and in about 20% of patients the INR is not in the target range prior to the procedure.

#### NOAC

After the introduction of NOACs many observational studies have been conducted to describe experiences with these new agents in the setting of ablation for AF [44]. Especially the timing of holding and resumption of these new drugs was largely unknown. Given its direct onset of effect, timing of resumption may affect postprocedural bleeding. Detailed analyses of the observational studies have confirmed this, showing that the studies with the most marked rates of bleeding and tamponade were characterised by a very short NOAC-free interval. Appreciating that heparin is administered during the procedure, and the initially unknown effects of combined heparin and NOAC use, many of the first studies used a regimen of interruption of NOAC. The first observational evidence suggested numerically, though not statistically significantly higher rates of thrombo-embolic events for interrupted NOAC regimens, with similar or somewhat lower bleeding rates when compared with warfarin regimens [45]. Meta-analyses on all observational data comparing interrupted regimens of NOAC versus VKA showed no difference in thrombo-embolic events and bleeding [45]. With increasing familiarity with the new drugs, uninterrupted periprocedural NOAC use has become a more accepted strategy. For a fair comparison between NOAC and VKA with regard to bleeding complications, both should be used in a setting of uninterrupted anticoagulation. This is in contrast to a recent, randomised Asian study that showed lower bleeding rates in case of interrupted NOAC use versus continued use of VKA [46]. The available observational studies that compared continued periprocedural use of NOACs with uninterrupted VKA reported similar efficacy and safety [45]. Randomised controlled trials were eagerly awaited. Based on the reported rates of periprocedural stroke in the meta-analyses, it should be acknowledged that—a priori—these trials were not powered to address this endpoint. Therefore, the emphasis shifted towards ‘safety and feasibility’, although it should be appreciated that some of the trials have been criticised for the design and power to address major bleeding as well. As of yet, two trials are currently recruiting. First, the ELIMINATE (A prospective, randomised, open-label, blinded endpoint evaluation parallel group study comparing edoxaban versus VKA in subjects undergoing catheter ablation of non-valvular atrial fibrillation; NCT02942576) study in which uninterrupted anticoagulation with edoxaban versus VKA is the topic of interest. The trial will not be performed in the Netherlands and France due to alleged issues with the study design. However, calculations based upon the RE-CIRCUIT trial demonstrate that for the endpoint bleeding, power should not be an issue. The AXAFA trial has addressed the same study question for apixaban [9, 47]. The first randomised, though somewhat small study (*n* = 248) of NOACs versus VKA was VENTURE-AF, in which rivaroxaban 20 mg once daily resulted in similar bleeding rates as uninterrupted VKA [41]. The largest trial so far (*n* = 635) has been performed with dabigatran, where patients in the RE-CIRCUIT trial had significantly higher bleeding rates on VKA than on uninterrupted NOAC [42].

Just recently, an updated meta-analysis on 12 studies, with in total 4962 patients, suggested similar rates of thrombo-embolism (NOAC 0.08% vs. VKA 0.16%; OR 0.66, 95% CI 0.19–2.30) and demonstrated significantly lower bleeding rates (NOAC 0.9% vs. VKA 2.0%; OR 0.50, 95% CI 0.30–0.84, *p* < 0.01) when uninterrupted use of NOACs was compared with uninterrupted use of VKA, in observational and randomised trials taken together [44]. The first evidence on interrupted NOAC regimens looks promising with regard to safety, but additional information with regard to the embolic protection is warranted [48].

### Peri-ablation anticoagulation—daily practice

In a 2014 European Heart Survey on periprocedural anticoagulation about 50–60% reported to use VKA prior to the procedure, with uninterrupted periprocedural VKA in 80%. In about 10% of procedures interrupted VKA with bridging was the periprocedural regimen. This latter strategy was less frequently observed in high volume centres and university hospitals [43].

Notably, a strategy of uninterrupted VKA has the logistic drawback that about 20% of patients will not have adequate INR control in the weeks prior to the procedure. Data on procedures performed in case of inadequate INR control prior to the procedure is scarce, although it might be considered in patients with a low risk for stroke. Procedural anticoagulation is secured with heparin administration that is driven by activated clotting time, but postprocedural anticoagulation coverage may be suboptimal. Appreciating the elective nature of the procedure, primarily focused on comfort and complaints and not on prognosis, inadequate INR control before the procedure may therefore result in postponement of the procedure.

In analogy to the planning of cardioversions for AF, NOACs have the advantage of predictable planning of the procedures. Currently, 30% of patients in Europe now receive a NOAC prior to the ablation. In half, the use is uninterrupted or just briefly interrupted. Strikingly, in the other half of cases an interruption of more than 2 days is reported, which results in suboptimal periprocedural coverage. This shows that more information is needed to guide the clinical approach of periprocedural use of NOACs [43].

At present, most of the available randomised and observational studies apply to dabigatran, followed by studies on experiences with rivaroxaban [48]. Uninterrupted regimens guarantee continuous anticoagulation, providing optimal protection against thrombo-embolic events. As for dabigatran, taken twice daily, the protocol of the RE-CIRCUIT study stated that the morning dose was not to be omitted before the ablation. Resumption after the procedure was at the same day, with a minimum of 3 hours after sheath removal [42]. For the once daily regimen of rivaroxaban, evening dosing was recommended and the factor Xa inhibitor could be re-initiated 6 hours after haemostasis was established [41].

These differences between these approaches demonstrate how important it is to define local protocols, both pre-ablation and post-ablation. If a regimen of uninterrupted anticoagulation is preferred, the NOACs with a once daily administration deserve special attention with regard to the time of administration (evening dosing). A short preprocedural interval of NOAC interruption will not cause much harm, given the often low CHA_2_DS_2_-VASc score of most patients undergoing ablation therapy [38, 42]. However, intervals of >2 days as reported in the recent survey should be avoided, not only in patients in AF prior to the procedure, but also in other patients to ensure a postprocedural antithrombotic coverage in the first few hours. Another important aspect concerns compliance. Whereas INR control provided an indication of the quality of preprocedural anticoagulation with VKA, in the NOAC era the patient is our only source. It should be noted that prior to the procedure the risk of thrombo-embolic events is extremely low for many patients in the target population, which may explain that about 10% of patients in the survey did not have any anticoagulation prior to the procedure [43]. Importantly, the proper level of anticoagulation during the procedure can always be achieved by administering heparin. However, to prevent procedure-related thrombo-embolic events it is of the utmost importance to keep the NOAC free interval after the procedure as short as possible. On the other hand, the interval should be long enough to ensure haemostasis at puncture sites.

In summary, there is a lot of experience and randomised evidence on strategies of uninterrupted use of VKA, with clear superiority in terms of major bleeding when compared to interrupted VKA and heparin bridging. In addition, thrombo-embolic events on the uninterrupted regimen of VKA are very low. In daily practice, the implementation of uninterrupted VKA seems satisfactory.

In contrast, centres using NOACs often fail to follow the approach as tested in the limited available randomised trials, which calls for attention to improve our daily practice. With an uninterrupted regimen, evening dosing is recommended for rivaroxaban. In case of dabigatran the morning dose does not need to be skipped. Resumption after the procedure is recommended 3–5 hours after haemostasis has been established. As far as the post-ablation period is concerned, at least 2 months of postprocedural anticoagulation is warranted. Long-term anticoagulation should be continued as per the original indication for subjects with risk factors for stroke, based on the CHA_2_DS_2_-VASc score. Data on the other NOACs for the indication of ablation are eagerly awaited, as well as additional evidence for interrupted regimens.
